# MICP-Treated Coral Aggregate and Its Application in Marine Concrete

**DOI:** 10.3390/ma18153619

**Published:** 2025-08-01

**Authors:** Rui Xu, Baiyu Li, Xiaokang Liu, Ben Peng, Guanghua Lu, Changsheng Yue, Lei Zhang

**Affiliations:** 1 School of Materials Science and Engineering, Tianjin Chengjian University, Tianjin 300384, China; xurui@tcu.edu.cn (R.X.);; 2 Tianjin Key Laboratory of Building Green Functional Materials, Tianjin Chengjian University, Tianjin 300384, China; 3 Central Research Institute of Building and Construction Co., Ltd., MCC Group, Beijing 100088, China

**Keywords:** coral aggregate, microbial-induced carbonate precipitation, mineralization mechanism, marine concrete, concrete interfacial transition zone

## Abstract

In marine engineering applications, substituting conventional crushed stone coarse aggregates with coral aggregates offers dual advantages: reduced terrestrial quarrying operations and minimized construction material transportation costs. However, the inherent characteristics of coral aggregates—low bulk density, high porosity, and elevated water absorption capacity—adversely influence concrete workability and mechanical performance. To address these limitations, this investigation employed microbial-induced carbonate precipitation (MICP) for aggregate modification. The experimental design systematically evaluated the impacts of substrate concentration (1 mol/L) and mineralization period (14 days) on three critical parameters, mass gain percentage, water absorption reduction, and apparent density enhancement, across distinct particle size fractions (4.75–9.5 mm, 9.5–20 mm) and density classifications. Subsequent application trials assessed the performance of MICP-treated aggregates in marine concrete formulations. Results indicated that under a substrate concentration of 1 mol/L and mineralization period of 14 days, lightweight coral aggregates and coral aggregates within the 4.75–9.5 mm size fraction exhibited favorable modification effects. Specifically, their mass gain rates reached 11.75% and 11.22%, respectively, while their water absorption rates decreased by 32.22% and 34.75%, respectively. Apparent density increased from initial values of 1764 kg/m^3^ and 1930 kg/m^3^ to 2050 kg/m^3^ and 2207 kg/m^3^. Concrete mixtures incorporating modified aggregates exhibited enhanced workability and strength improvement at all curing ages. The 28-day compressive strengths reached 62.1 MPa (11.69% increment), 46.2 MPa (6.94% increment), and 60.1 MPa (14.91% increment) for the 4.75–9.5 mm, 9.5–20 mm, and continuous grading groups, respectively, compared to untreated counterparts.

## 1. Introduction

The development of islands and reefs plays a pivotal role in facilitating regional resource utilization and strategic infrastructure deployment. As the primary construction material, conventional concrete requires aggregate components constituting 70–80% of its total mass [1,2,3], creating substantial aggregate requirements for large-scale marine infrastructure projects. However, traditional concrete applications face dual constraints in marine engineering: limited availability of terrestrial mineral resources and prohibitive transportation costs associated with continental material sourcing. These challenges necessitate the adoption of alternative materials. Numerous studies have shown that preparing coral aggregate concrete (CAC) using waste coral fragments from river excavation, natural weathering, and port dredging poses no threat to natural ecosystems, while reducing energy consumption, carbon dioxide emissions, and transportation costs [4,5,6,7]. Therefore, local utilization of coral aggregates represents an essential strategy for the construction of marine islands.

Coral formations originate from biogenic accretion processes wherein coral polyps sequester dissolved calcium and carbonate ions from seawater to construct calcium carbonate exoskeletons. Through diagenetic compaction spanning millennia, these biological structures lithify into porous limestone formations. Mineralogically, these formations comprise metastable calcium carbonate polymorphs (vaterite and aragonite) and stable calcite phases, with a chemical composition dominated by calcium carbonate (CaCO_3_) at 96% purity [8,9]. Characteristic physical parameters include bulk density (900 kg/m^3^), apparent density (1800 kg/m^3^), and porosity exceeding 50%, with 1 h water absorption rates surpassing those of ceramic coarse aggregates by 16%. Unlike conventional mineral aggregates, coral-derived materials exhibit distinct structural characteristics: low specific gravity, interconnected pore networks, and surface contamination from marine sediments (clay/silt adhesion) during harvesting operations [10,11]. These inherent properties directly impact cementitious composites, manifesting as reduced workability during placement, compromised constructability, and diminished compressive strength development in coral aggregate concretes.

Contemporary research on coral aggregate enhancement primarily focuses on two methodological approaches, physical densification and chemical surface modification, based on the mineralogical and physical characteristics of marine-derived materials. Liu et al. [12] demonstrated that granular blast furnace slag (GBFS) and sodium silicate (SS) effectively fill coral aggregate pores, reducing water absorption and the crushing index by over 50%. Tan et al. [13] found that nano-TiO_2_ enhanced cement hydration by filling matrix pores to form a denser structure, increasing coral concrete compressive strength by 22.2%. Chu et al. [14] achieved a 35.3% improvement in coral concrete workability through citric acid cleaning and basic magnesium sulfate cement coating, with flexural and compressive strength rising by 20.5% and 31.8%, respectively. Liu et al. [15] reported that silane coupling agents (organic solvents) significantly reduce coral coarse aggregate water absorption, promote chemical bonding between the aggregate and cement mortar, and enhance interfacial adhesion. Although physical and chemical modification methods demonstrate promising results, they exhibit three key limitations. First, physical fillers penetrate aggregate interiors unevenly, which limits their effectiveness at greater depths. Second, nanoparticles incur high material costs and show poor economic feasibility. Third, chemical treatments such as acid solution or silane coupling agents face dosage control challenges; inaccurate quantities may weaken aggregate strength while introducing harmful ions into concrete.

To mitigate these limitations, microbial-induced carbonate precipitation (MICP) has emerged as a sustainable modification approach, offering environmental benefits and material compatibility advantages [16,17,18,19]. Research demonstrates that microorganisms serve as nucleation sites for biomineralization processes, enzymatically catalyzing calcium carbonate crystallization through metabolic pathways. The precipitated calcium carbonate effectively fills material defects [20,21,22]. Zhang et al. [23] reported that *Bacillus mucilaginosus* treatment enhanced artificial aggregate properties, achieving a 2620 kg/m^3^ apparent density (vs. 2520 kg/m^3^ untreated) and 4.8% water absorption (vs. 9.7% untreated), confirming that MICP improves aggregate densification. Guan et al. [24] employed *Bacillus pasteurii* to modify coal gangue aggregate, achieving 18.5% and 24.1% reductions in water absorption and the crushing index, respectively, with a concurrent 8.13% increase in the 28-day compressive strength of coal gangue concrete. Li et al. [25] treated recycled brick aggregates through soaking in *Bacillus subtilis* and activated sludge mixed bacteria, resulting in 20% and 8.1% decreases in water absorption and the crushing index, respectively, along with 16.7% and 11.7% enhancements in compressive and tensile strength of the resulting concrete. Collectively, these studies demonstrate the feasibility of microbial aggregate modification. However, research on microbial modification of coral aggregates, especially investigations into their underlying mechanisms, remains inadequately studied. The mechanism of microbial-modified coral aggregate has not been fully revealed. Furthermore, coral aggregates exhibit significant density variations, yet limited research exists regarding modifying aggregates across different density ranges.

This work employed MICP technology for aggregate modification. *Bacillus pasteurii* was selected as the biomineralization agent due to its superior carbonate precipitation efficacy. The influence of substrate concentration and mineralization period on the modification efficacy of coral aggregates with varying bulk densities and particle size fractions was investigated through soaking in bacterial solution and batch-wise calcium source addition. Treatment outcomes were quantified via mass gain percentage, water absorption rate, and apparent density. The MICP-treated coral aggregates were subsequently incorporated into marine concrete matrices, with workability parameters and compressive strength development evaluated. Microstructural characterization of cement–aggregate interfaces and biogenic precipitates was conducted using scanning electron microscopy (SEM), ultra-depth field microscopy, and X-ray diffractometry (XRD).

## 2. Materials and Methods

### 2.1. Raw Materials

*Bacillus pasteurii* (obtained from the German Collection of Microorganisms and Cell Cultures) was used. Based on the existing literature [26,27,28], the culture medium for *Bacillus pasteurii* consisted of beef extract (3 g/L), peptone (5 g/L), and deionized water, while the mineralized substrate consisted of urea and calcium acetate mixed in a 1:1 ratio, which was prepared and set aside after incubating *Bacillus pasteurii* for 1 day. Beef extract, peptone, urea and calcium acetate were purchased from Tianjin Guangfu Technology Development Co., Ltd., Tianjin, China.

**Figure 1:**
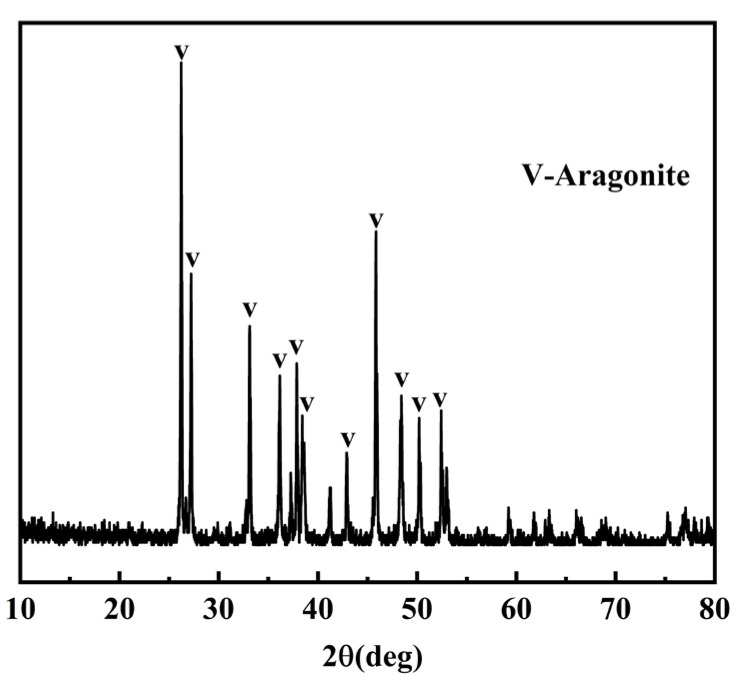
XRD patterns of coral aggregates.

The coral aggregate used in this study was sourced from Dehao Mineral Products Processing Plant, Lingshou County, Shijiazhuang City, Hebei Province, China. The particle size distribution, XRD pattern, and chemical composition of the coral aggregate are presented in Table 1, Figure 1, and Table 2, respectively. P·O 42.5 grade cement was used as the cementitious material. The natural fine aggregate was natural river sand, and class I fly ash and grade I silica fume were used. The chemical compositions of cement, fly ash, and silica fume are presented in Table 3. The admixture was a polycarboxylate superplasticizer with a water reduction rate of up to 25%.

### 2.2. Sample Preparation and Experimental Methods

#### 2.2.1. Treatment and Experimental Methods for Coral Aggregates

The coral aggregate was mechanically sieved, washed, and dried to obtain three particle size fractions: 4.75–9.50 mm, 9.50–20 mm, and 20–26 mm. After cleaning, the chloride ion content of the coral aggregate decreased from 0.3 % to 0.018 %. It meets the requirement of T/CECS 10090-2020 Coral aggregate used for concrete that the chloride ion content in coral aggregates should be less than or equal to 0.02% [29]. The physical properties by particle size are shown in Table 4. Coral aggregates with a 9.50–20 mm particle size were classified as lightweight coral aggregates (<1800 kg/m^3^) and heavyweight coral aggregates (>2050 kg/m^3^) based on apparent density difference, following the method of Su et al. [30]. Coral aggregates not classified by apparent density were designated as composite coral aggregates. The physical properties with varying bulk densities are shown in Table 5.

First, the effect of substrate concentration on modified coral aggregate properties was investigated. Substrate concentrations of 0.2 mol/L, 0.5 mol/L, 1.0 mol/L, 1.5 mol/L, and 2.0 mol/L were tested using light, heavy, and composite coral aggregates with a 9.5–20 mm particle size at a fixed mineralization period of 3 days. Next, the effect of mineralization period on MICP-modified coral aggregate was investigated at the optimal substrate concentration. Mineralization periods of 3, 7, 14, 21, and 28 days were evaluated using light, heavy, and composite coral aggregates with a 9.5–20 mm particle size. Finally, coral aggregates of different particle sizes (4.75–9.5 mm, 9.5–20 mm, and 20–26 mm) were modified under optimal substrate concentration and mineralization period conditions.

**Figure 2:**
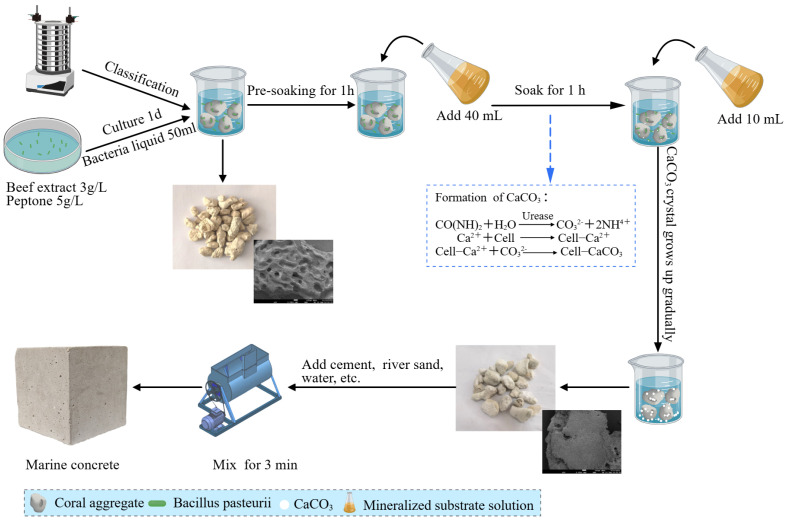
Flowchart of MICP treatment for coral aggregates and marine concrete preparation.

The modification method employed soaking treatment, with the process mechanism illustrated in Figure 2. The specific steps are as follows: (1) The bacterial solution was cultured for 1 day, and the mineralization solution was prepared. (2) Separated coral aggregates (≈40 g per group) were placed in a mineralization container. Then, 50 mL of bacterial solution was added to ensure the solution level exceeded the aggregate surface, followed by 1 h of pre-soaking to facilitate microorganism penetration via capillary absorption. (3) An amount of 40 mL of mineralized substrate solution was added for 6 h of soaking. (4) The remaining 10 mL of mineralized substrate solution was added. The batch-wise addition ensured enhanced deposition of mineralization products on the aggregates [31,32].

#### 2.2.2. Preparation and Experimental Method of Coral Concrete

In this experiment, the lightweight aggregate concrete preparation sequence without pre-wetting treatment specified in JGJ51-2002 Technical Specification for Lightweight Aggregate Concrete was used for coral concrete preparation [33].

Coral concrete mix proportion design is complex due to the slurry enrichment coefficient. To simplify the experimental content and highlight the research objectives, the mix proportion parameters established by Wu [34] were adopted. The modification conditions for coral aggregate were as follows: substrate concentration of 1 mol/L and mineralization period of 14 days. Mix proportions are shown in Table 6.

### 2.3. Experimental Characterization

#### 2.3.1. Water Absorption and Apparent Density of Coral Aggregate

Water absorption and apparent density were measured according to GB/T17431.2-2010 Lightweight Aggregates and Its Test Methods-Part 2: Test Methods for Lightweight Aggregates [35].

#### 2.3.2. Deposition Efficiency

The deposition efficiency is used as the technical index of substrate concentration, and the calculation method is shown in Formula (1).

(1) $$u=\frac{m}{cVM}\times 100\%$$

*u*—Deposition efficiency of mineralized solution (%);

*m*—Sediment yield (g);

*c*—Substrate concentration in mineralized solution (mol/L);

*V*—Mineralized liquid volume (L);

*M*—Molar mass of calcium carbonate (g/mol).

#### 2.3.3. Basic Properties of Coral Concrete

(1) Workability of fresh concrete

Concrete workability is typically determined by slump. However, the concrete in this study exhibited slump values exceeding 160 mm, indicating large-flow concrete. Therefore, the slump/slump flow diameter ratio was used to comprehensively evaluate workability. When this ratio approaches 0.4, the concrete demonstrates no segregation, no bleeding, good cohesion, and optimal workability [36,37,38].

(2) Mechanical behavior of concrete

Mechanical properties were tested in accordance with GB/T 50081-2002 Standard for Test Method of Mechanical Properties on Ordinary Concrete [39].

#### 2.3.4. Mineral Composition Analysis

Mineralized products were ground into powder, dried at 80 °C to constant mass, and then analyzed by XRD (D/MAX-UItima Ⅳ, Rigaku Corporation, Tokyo, Japan) to determine the phase composition.

#### 2.3.5. Morphology Analysis

Surface and cross-sectional deposits on coral aggregate before and after modification were observed using ultra-depth field microscopy (VHX-600e, Keyence Corporation, Osaka, Japan). Internal mineralization products and concrete interfacial transition zones were examined using SEM (JSM-7800F, Kabushiki Kaisha, Tokyo, Japan).

## 3. Results

### 3.1. MICP-Treated Coral Aggregates with Single Particle Size Fraction

#### 3.1.1. Weight Gain Rate

**Figure 3:**
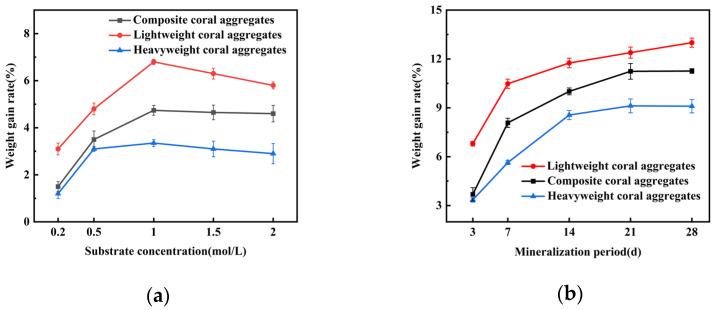
Weight gain rate of MICP-treated coral aggregates: (**a**) substrate concentration dependence; (**b**) mineralization duration effect.

Figure 3 illustrates the effect of MICP treatment on the weight gain rate for three coral aggregate types. Figure 3a shows that the weight gain rate increased initially and then decreased slightly with increasing substrate concentration. Peak gains of 6.8%, 4.74%, and 3.35% occurred at 1.0 mol/L for lightweight, composite, and heavyweight aggregates, respectively, with a 3.45% difference between lightweight and heavyweight aggregates. Figure 3b shows time-dependent trends under the optimal substrate concentration (1.0 mol/L), where mass accumulation accelerated during the first 14 days. Maximum gains of 11.75%, 10.01%, and 8.56% were achieved by lightweight, composite, and heavyweight aggregates at 14 days. The observed results occur because lightweight coral aggregate has the highest porosity, whereas heavy coral aggregate has the lowest. As a result, lightweight aggregate provides a larger specific surface area, enhancing bacterial adsorption capacity and improving the modification effectiveness.

#### 3.1.2. Water Absorption

**Figure 4:**
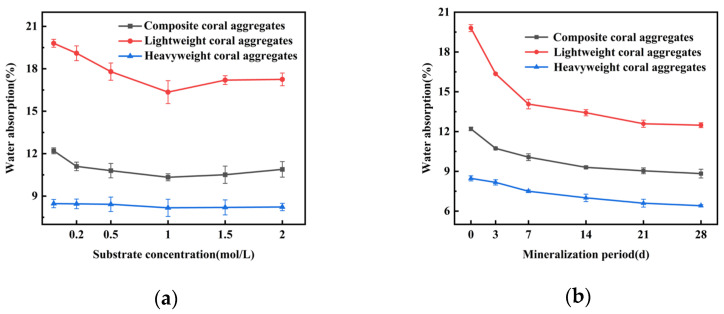
Water absorption of MICP-treated coral aggregates: (**a**) substrate concentration dependence; (**b**) mineralization duration effect.

Figure 4 illustrates the impact of MICP treatment on water absorption for three coral aggregate types. As shown in Figure 4a, increasing substrate concentration reduced water absorption rates, with the most pronounced effect at 1 mol/L. Specifically, lightweight coral aggregates exhibited a decline from 19.8% to 16.35% (17.42% reduction), composite coral aggregates decreased from 12.2% to 10.33% (15.32% reduction), and heavyweight coral aggregates dropped from 8.47% to 8.17% (3.54% reduction). However, at 1.5 mol/L, water absorption rates increased slightly. Figure 4b shows that under the 1.0 mol/L substrate concentration, water absorption rates decreased progressively with mineralization duration. Notably, the most rapid reduction occurred within the first 14 days.

#### 3.1.3. Apparent Density

**Figure 5:**
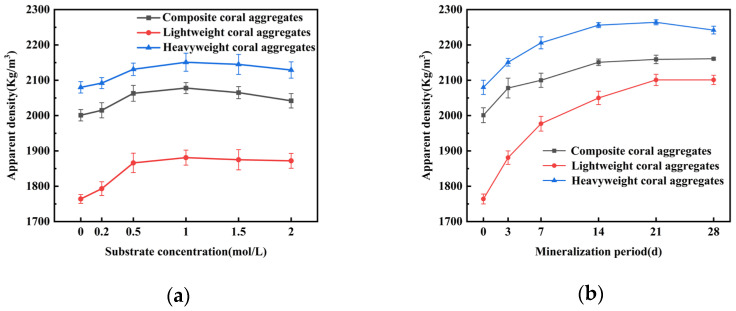
Apparent density of MICP-treated coral aggregates: (**a**) substrate concentration dependence; (**b**) mineralization duration effect.

Figure 5 reveals the influence of MICP treatment on the apparent density of three coral aggregate types. As shown in Figure 5a, apparent density exhibited a biphasic response to substrate concentration, peaking at 1 mol/L with increases from 1764 kg/m^3^ to 1881 kg/m^3^ (6.63% increment), 2001 kg/m^3^ to 2078 kg/m^3^ (3.85% increment), and 2080 kg/m^3^ to 2151 kg/m^3^ (3.41% increment) for lightweight, composite, and heavyweight aggregates, respectively. Density decreased at concentrations exceeding 1 mol/L. Figure 5b shows a positive correlation between mineralization duration and apparent density at 1.0 mol/L. After 14 days of mineralization, density increased from 1764 kg/m^3^ to 2050 kg/m^3^ (16.21% increment), 2001 kg/m^3^ to 2151 kg/m^3^ (7.50% increment), and 2080 kg/m^3^ to 2256 kg/m^3^ (8.46% increment) for each aggregate type.

Considering engineering efficiency and cost-effectiveness, the optimal MICP parameters were established as a 1.0 mol/L substrate concentration and 14-day mineralization duration, with lightweight aggregates exhibiting superior performance enhancement.

### 3.2. MICP-Treated Coral Aggregates with Different Particle Size Fractions

**Figure 6:**
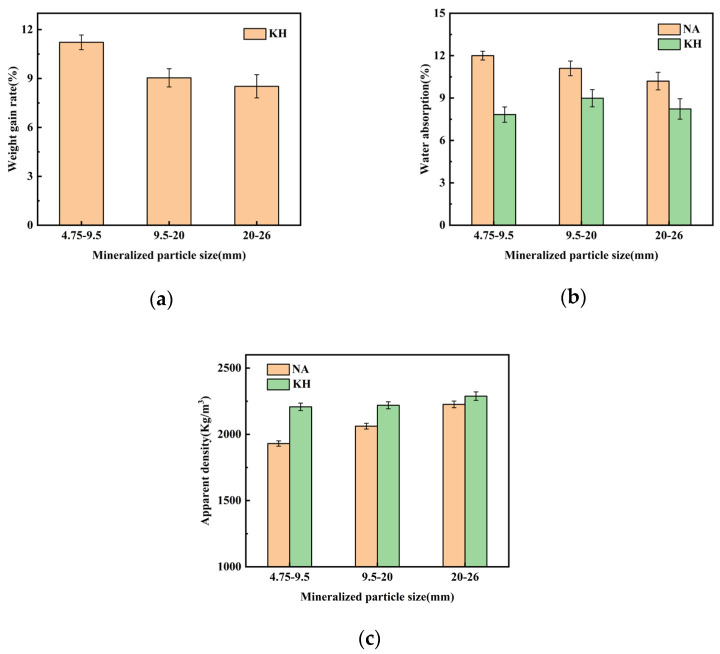
Modification effects on physical properties of MICP-treated coral aggregates by particle size fraction: (**a**) weight gain rate; (**b**) water absorption; (**c**) apparent density (NA: non-mineralized aggregates; KH: mineralized aggregates).

Figure 6 illustrates the effects of MICP modification on coral aggregates across particle size fractions, where NA denotes untreated aggregates and KH indicates biomineralized counterparts. Figure 6a reveals an inverse correlation between particle size and weight gain rate, with increases of 11.22%, 9.04%, and 8.52% for 4.75–9.5 mm, 9.5–20 mm, and 20–26 mm aggregates, respectively. This indicates enhanced biomineralization efficiency in smaller aggregates. Figure 6b demonstrates significant reductions in water absorption across all size fractions after treatment: (1) 4.75–9.5 mm: 12% to 7.83% (34.75% reduction); (2) 9.5–20 mm: 11.1% to 8.99% (19% reduction); and (3) 20–26 mm: 10.2% to 8.23% (19.31% reduction). The 4.75–9.5 mm aggregates exhibited the greatest reduction. Figure 6c shows that MICP treatment affects apparent density analogously to weight gain rate. Post-treatment apparent density increased: (1) 4.75–9.5 mm: 1930 kg/m^3^ to 2207 kg/m^3^ (14.35% increment); (2) 9.5–20 mm: 2061 kg/m^3^ to 2219 kg/m^3^ (7.66% increment); and (3) 20–26 mm: 2126 kg/m^3^ to 2284 kg/m^3^ (7.43% increment). The 4.75–9.5 mm aggregates achieved the greatest density enhancement. Thus, 4.75–9.5 mm coral aggregates exhibited the most comprehensive performance improvement under optimal conditions. This occurs because smaller aggregates (4.75–9.5 mm) have higher porosity and larger specific surface area, facilitating bacteria adsorption and biogenic CaCO_3_ deposition. Additionally, their smaller pores are more readily filled by biogenic CaCO_3_.

### 3.3. Application of MICP-Treated Coral Aggregates in Marine Concrete

#### 3.3.1. Concrete Workability

Table 7 presents workability variations of coral aggregate concrete before and after biomineralization treatment. Table 7 shows that the slump and slump flow diameter of concrete with 4.75–9.5 mm aggregates were lower than those of other aggregate types due to higher porosity and water absorption in smaller aggregates, which rapidly absorb mixing water and reduce fresh concrete fluidity. After treatment, slump increased by 35 mm, 13 mm, and 28 mm for 4.75–9.5 mm, 9.5–20 mm, and continuously graded aggregates, respectively, while slump flow diameter increased by 122 mm, 58 mm, and 119 mm. These results indicate that MICP treatment differentially enhances workability, with particularly significant improvements in 4.75–9.5 mm aggregate concrete. The slump-to-slump flow diameter ratio exceeded 0.42 for single-size-fraction concrete (4.75–9.5 mm and 9.5–20 mm), whereas continuously graded concrete exhibited ratios near 0.4. Post-treatment, all gradations showed ratio convergence toward 0.4.

#### 3.3.2. Concrete Compressive Strength

Table 8 demonstrates improved 3-day, 7-day, and 28-day compressive strength for all aggregate types after treatment. The 4.75–9.5 mm fraction exhibited the highest strength, with 28-day strength increasing from 55.6 MPa (pre-treatment) to 62.1 MPa (post-treatment). Furthermore, 28-day strength increased by 6.5 MPa, 3 MPa, and 7.8 MPa for 4.75–9.5 mm, 9.5–20 mm, and continuous gradation aggregates, respectively, after treatment, representing relative increases of 11.69%, 6.94%, and 14.91% versus pre-treatment values. The continuously graded group showed the greatest strength enhancement.

## 4. Discussion

### 4.1. Deposition Efficiency

Table 9 presents the deposition efficiency at different substrate concentrations. The data indicate that deposition efficiency increases and then decreases with rising substrate concentration, peaking at 1 mol/L. This trend occurs because elevated urea concentrations stimulate microbial urease activity below 1 mol/L, enhancing carbonate deposition. Conversely, concentrations exceeding 1 mol/L inhibit urease activity due to calcium ion toxicity and microbial adsorption by positively charged ions, reducing efficiency [40,41]. Consequently, a 1 mol/L substrate concentration yields optimal mineralization for coral aggregate modification.

### 4.2. Apparent Morphology

**Figure 7:**
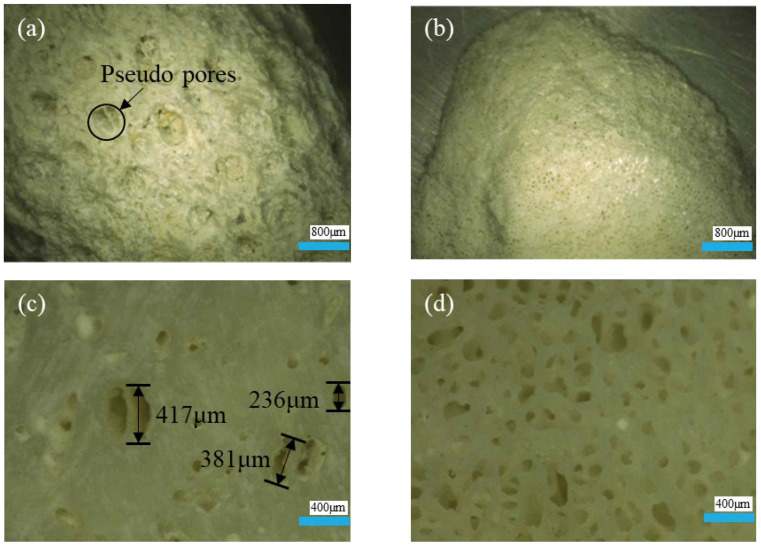
Surface and cross-sectional morphology of coral aggregates: (**a**) heavyweight coral aggregate surface; (**b**) lightweight coral aggregate surface; (**c**) heavyweight coral aggregate cross-section; (**d**) lightweight coral aggregate cross-section.

Figure 7 compares the surface and cross-sectional morphologies of heavy- and lightweight coral aggregates. As shown in Figure 7a, heavyweight coral aggregates exhibit few large surface pores, primarily isolated pseudo-pores unconnected to internal structures, resulting in irregular surfaces. This accounts for their minimal water absorption. Conversely, Figure 7b shows that lightweight coral aggregates feature a honeycomb-like structure with numerous interconnected micropores forming continuous exterior–interior networks, explaining their higher water absorption. Cross-sectional analysis (Figure 7c,d) reveals distinct differences. Heavy aggregates exhibit cross-sectional porosity exceeding surface porosity, but pores are non-uniformly distributed and dominated by macropores (>200 μm), with limited total internal porosity. Light aggregates show morphological consistency between the surface and cross-section, characterized by smaller pore diameters, complex interconnected pore networks, thin pore walls, and higher total porosity with a larger specific surface area. These morphological differences explain superior modification efficacy in lightweight coral aggregates: (1) enhanced bacterial adsorption facilitates biogenic CaCO_3_ deposition on surfaces; (2) interconnected micropores are more readily filled due to smaller dimensions.

**Figure 8:**
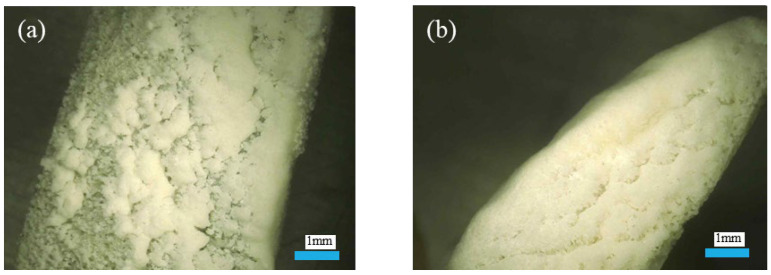
The cross-section deposition of coral aggregate after MICP modification for 14 d. (**a**) Heavyweight coral aggregate cross-section; (**b**) lightweight coral aggregate cross-section.

Figure 8 compares the cross-sectional deposition patterns of heavyweight and lightweight coral aggregates. The results demonstrate that lightweight aggregates exhibit greater sediment deposition than heavyweight aggregates, with their cross-sections being predominantly filled with biogenic CaCO_3_. This phenomenon occurs because lightweight aggregates possess a higher porosity and specific surface area, resulting in greater bacterial loading capacity. Consequently, they mineralize more CaCO_3_ than heavyweight aggregates during MICP. Additionally, initial reaction stages produce abundant free CaCO_3_ in solution, which gradually deposits over time. Due to their enhanced porosity and surface area, lightweight aggregates adsorb this free CaCO_3_ more effectively than heavyweight aggregates. This also significantly contributes to the superior mineralization efficacy of lightweight aggregates.

### 4.3. Phase Composition of Mineralization Products

**Figure 9:**
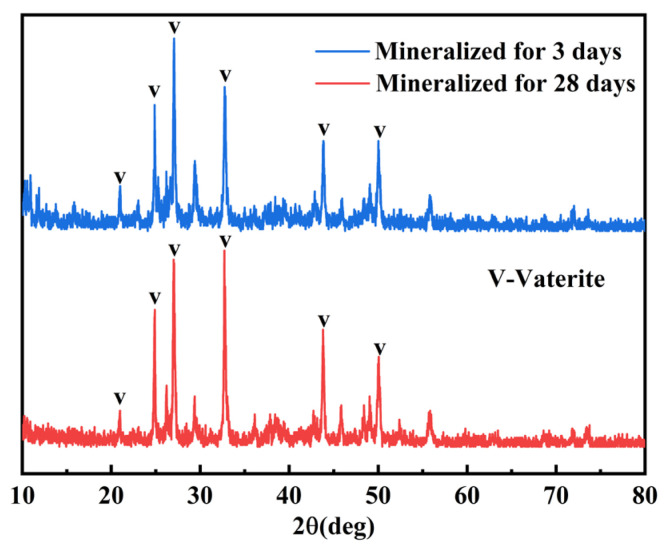
XRD patterns of surface deposits on coral aggregates after different mineralization periods.

Figure 9 shows XRD patterns of surface powder from coral aggregates after 3-day and 28-day mineralization periods. The results indicate that deposition products are CaCO_3_ (vaterite). This confirms that biogenic CaCO_3_ does not undergo crystal transformation during its growth process, even with extended mineralization periods. Comparative analysis of Figure 1 and Figure 9 reveals that the calcium carbonate produced by microbial mineralization deposition is predominantly vaterite, a metastable polymorph characterized by fine crystalline particles and higher surface energy compared to aragonite. This phyicochemical property enhances its reactivity with other concrete constituents, leading to reduced porosity and improved compactness as well as mechanical strength [42].

### 4.4. Microstructure

**Figure 10:**
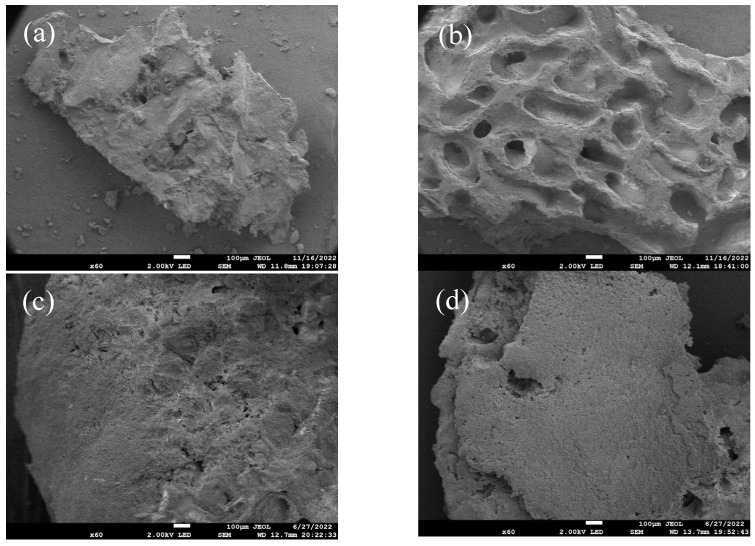
SEM images of coral aggregates before and after modification. (**a**) Unmodified heavyweight coral aggregates; (**b**) unmodified lightweight coral aggregates; (**c**) heavyweight coral aggregates modified for 14 d; (**d**) lightweight coral aggregates modified for 14 d.

Figure 10 illustrates the surface morphological evolution of coral aggregates before and after mineralization treatment. Heavyweight coral aggregates show dense, low-porosity structures (Figure 10a,b). This microstructure explains their low water absorption and high strength. Modified lightweight coral aggregates display complete mineral coverage (Figure 10d). Mineral deposits fully interconnect, sealing nearly all surface pores. This confirms successful biomineralization. Modified heavyweight aggregates exhibit incomplete mineral layers (Figure 10c). Gaps remain between sediment patches, leaving some pores open. Lightweight aggregates therefore achieve better mineralization than heavyweight ones. However, the sediment is not flatly connected as a whole, and there are still some pores on the aggregate surface that are not completely blocked. This is also the reason why the mineralization modification effect of lightweight coral aggregate is better than that of heavy coral aggregate.

**Figure 11:**
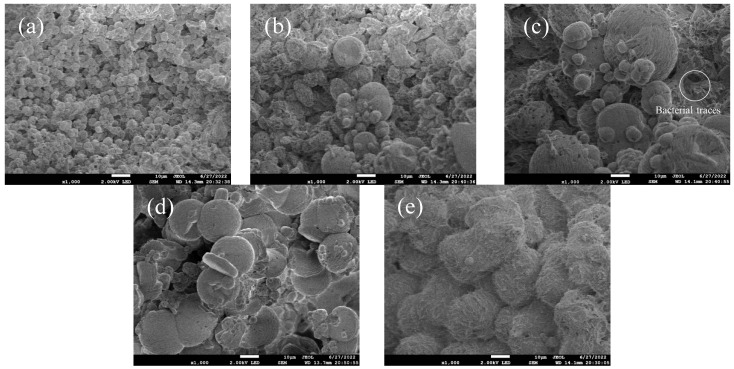
SEM images of surface deposits on MICP-treated coral aggregates at different mineralization periods ((**a**): 3-day; (**b**): 7-day; (**c**): 14-day; (**d**): 21-day; (**e**): 28-day).

Figure 11a–e show scanning electron microscopy images of surface deposits on coral aggregates after 3, 7, 14, 21, and 28 days of mineralization. Figure 11a shows that after 3 days of soaking, mineralization had just ceased and deposits were in a loose spherical state—an initial morphology of biogenic CaCO_3_ post-treatment. Figure 11b–e reveal large spheres bonded to small spheres, occurring because spherical CaCO_3_ crystals grow with increasing soaking time, compressing and bonding to form cohesive structures that fill coral aggregate pores. In Figure 11e (28-day), minimal small spherical CaCO_3_ indicates near-complete growth with dense inter-crystal connections. Additionally, Figure 11c (14 days) shows rod-shaped pits in deposits. These pits which are consistent with Bacillus pasteurii’s rod-shaped morphology, likely representing bacterial imprints. This demonstrates that microbial mineralization involves gradual deposition, with bacteria acting as nucleation sites. From Figure 11, it is evident that calcium carbonate crystals are fully formed and densely interconnected after 14 days of mineralization. Beyond this period, prolonged mineralization induces negligible changes in crystal morphology and microstructure. This structural stability explains why the modified coral aggregate exhibits minimal performance variations when treated for more than 14 days.

### 4.5. Modification Mechanism Analysis

*Bacillus pasteurii* is a ureolytic microorganism. Based on ureolytic mineralization mechanisms and prior physical/morphological analyses, the MICP modification mechanism for coral aggregates is analyzed below.

(1) Bacterial solution soaking stage

Coral aggregates are lightweight and porous, with a high-water absorption rate (1 h absorption reaches 90% of 24 h capacity). When submerged in bacterial solution, bacteria enter surface pores and cracks via fluid transport, adhering internally to prepare for mineralization.

(2) Mineralization reaction and deposition

Microorganisms hydrolyze urea to produce CO_3_^2−^ via intracellular/extracellular ion gradients. Cell surfaces contain abundant negative charges that attract free Ca^2+^, prompting immediate precipitation of CaCO_3_. Fewer bacteria inside aggregates mineralize CaCO_3,_ which fills smaller pores. Free bacteria in solution generate substantial CaCO_3_, which deposits in deeper pores and weak zones. However, excessive substrate concentration (>1 mol/L) inhibits microbial enzymes and increases urea concentration, causing surface pore clogging that prevents substrate penetration. This limits deep mineralization, reducing modification efficacy. Thus, an optimal substrate concentration (1 mol/L) is essential.

(3) Crystal growth stage

*Bacillus pasteurii* mineralization ceases by day 3, but surface deposits lack mechanical strength and exhibit week aggregate bonding, making them prone to detachment. Over time, water pressure within the solution compresses CaCO_3_ crystals, promoting growth and strength development. By day 14, deposits achieve near-complete surface coverage with dense pore/crack filling, yielding optimal modification.

### 4.6. Microstructure of Concrete Interface Transition Zone

**Figure 12:**
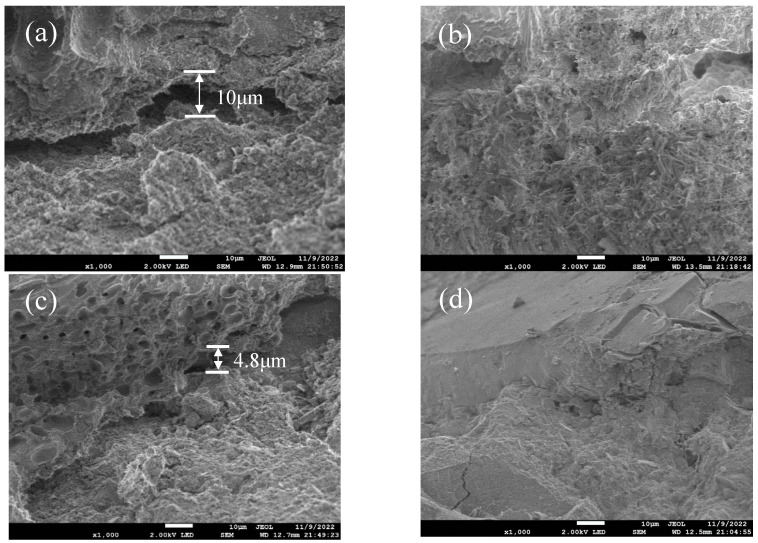
Interfacial transition zone in coral concrete: (**a**) untreated 9.5–20 mm aggregate; (**b**) ICP-treated 9.5–20 mm aggregate; (**c**) untreated 4.75–9.5 mm aggregate; (**d**) MICP-treated 4.75–9.5 mm coral aggregate.

The interfacial transition zone (ITZ) of coral concrete before and after mineralization was observed using scanning electron microscopy. Figure 12a shows the ITZ of concrete with untreated 9.5–20 mm coral aggregates, revealing obvious cracks (≈10 μm wide) sparsely filled by hydration products. Figure 12b demonstrates that MICP-treated aggregates had significantly reduced ITZ cracks and increased hydration product density. The ITZ of concrete with untreated 4.75–9.5 mm aggregates (Figure 12c) exhibited a narrower width and crack sizes <5 μm compared to 9.5–20 mm counterparts. Figure 12d shows that after MICP treatment, ITZ cracks were substantially narrowed, with abundant hydration products filling pores and cracks. The reason for these results is that untreated coral aggregates exhibit high water absorption, which depletes mixing water from the surrounding cement paste during concrete mixing. This depletion lowers the local water–cement ratio adjacent to the aggregates, delaying cement hydration in the ITZ. Consequently, the ITZ widens, compromising concrete strength. Following MICP treatment, reduced water absorption in coral aggregates minimizes water extraction from the cement paste. This preserves adequate local water–cement ratios near aggregate surfaces, enabling timely and complete cement hydration. The resulting denser, narrow ITZ enhances concrete strength.

## 5. Conclusions

In this work, coral aggregates were modified using *Bacillus pasteurii*, and the modified aggregates were incorporated into marine concrete. The main conclusions are as follows:

(1) By evaluating the weight gain rate, water absorption rate, and apparent density, optimal mineralization conditions were established as a 1 mol/L substrate concentration and 14-day mineralization period. Under these conditions, lightweight coral aggregates and 4.75–9.5 mm aggregates achieved superior modification: their weight gain rates reached 11.75% and 11.22% and their water absorption decreased by 32.22% and 34.75%, while their apparent densities increased from 1764 kg/m^3^ to 2050 kg/m^3^ and 1930 kg/m^3^ to 2207 kg/m^3^, respectively.
(2) XRD analysis confirmed vaterite as the exclusive CaCO_3_ polymorph in deposits. No crystal transformation occurred during mineralization. Ultra-depth microscopy and SEM revealed complete biogenic CaCO_3_ deposition in aggregate pores, with crystal size and inter-crystal connectivity increasing over the mineralization period.
(3) After MICP treatment, all concrete groups exhibited enhanced workability and age-dependent strength gains. The workability of the three concrete groups was significantly improved, and the strength of the modified concrete was increased at each age. The 28-day compressive strengths reached 62.1 MPa (11.69% increase) for the 4.75–9.5 mm group, 46.2 MPa (6.94% increase) for the 9.5–20 mm group, and 60.1 MPa (14.91% increase) for the continuously graded group. These improvements stem from denser aggregate–cement matrix bonding after MICP treatment, which eliminates ITZ cracks and optimizes interfacial structure.

This study elucidates the mechanism of MICP modification for coral aggregates and develops high-performance marine concrete. The findings provide a foundation for coral aggregate enhancement and advance marine infrastructure development, with significant applications in offshore and island construction. Future studies could explore spray-based modification to improve efficiency and economic sustainability. Another goal would be to study the long-term durability of coral aggregate concrete in the marine environment. Moreover, the protection of the marine ecological environment through promoting sustainable application of coral aggregate concrete in marine engineering will be the focus of future work as well.

## Figures and Tables

**Table 1:** Accumulated retained percentage of coarse coral aggregates.

Bore Diameter (mm)	26	20	9.5	4.75	2.36
The cumulative triage (%)	2.15	11.33	73.67	99	99.29

**Table 2:** Chemical properties of coral aggregate (wt.%).

Properties	CaO	SiO_2_	Fe_2_O_3_	Al_2_O_3_	MgO	LOI
Coral aggregate	52.69	1.7	0.6	0.36	0.78	41.3

**Table 3:** Chemical properties of the experiment materials (wt.%).

Properties	Al_2_O_3_	CaO	Fe_2_O_3_	K_2_O	MgO	Na_2_O	SO_3_	SiO_2_	TiO_2_
Cement	8.47	51.90	4.04	0.55	3.02	0.36	1.84	21.29	0.48
Fly ash	41.56	4.16	4.93	0.67	0.42	0.012	0.78	43.45	1.85
Silica fume	0.704	0.588	0.216	1.1	0.986	0.342	0.216	95.6	0

**Table 4:** Physical properties of coral aggregates by particle size range.

Particle Sizes (mm)	Porosity	Apparent Density	Water Absorption (%)	
	(%)	(kg/m^3^)	1 h	24 h
4.75–9.5	53.1	1930	12	12.9
9.5–20	50.1	2061	11.1	11.23
20–26	47.7	2126	10.2	10.3

**Table 5:** Physical properties of coral aggregates with varying bulk densities.

Bulk Densities	Porosity	Apparent Density	Water Absorption (%)	
	(%)	(kg/m^3^)	1 h	24 h
Lightweight	53.5	1764	19.8	19.9
Heavyweight	42.6	2080	8.47	8.51
Composite	50.1	2001	12.2	12.5

**Table 6:** Mix proportion design of coral aggregate concrete (kg/m^3^).

Grade	Cement	Silica Fume	Fly Ash	River Sand	Coral Aggregate	Water Consumption	Water Reducer
C50	600	75	75	688	586	225	37.5

**Table 7:** Workability of coral concrete by aggregate gradation (NA: non-mineralized aggregates; KH: mineralized aggregates).

Aggregate Gradation	4.75–9.5 mm (NA)	4.75–9.5 mm (KH)	9.5–20 mm (NA)	9.5–20 mm (KH)	Continuous Grading (NA)	Continuous Grading (KH)
Slump (mm)	210	245	257	270	230	258
Slump flow diameter (mm)	490	612	585	643	542	661
Slump/Slump flow diameter	0.43	0.40	0.44	0.42	0.42	0.39

**Table 8:** Compressive strength (MPa) of coral concrete by aggregate gradation and curing age (NA: non-mineralized aggregates; KH: mineralized aggregates).

Aggregate Gradation	4.75–9.5 mm (NA)	4.75–9.5 mm (KH)	9.5–20 mm (NA)	9.5–20 mm (KH)	Continuous Grading (NA)	Continuous Grading (KH)
3 d	42.2	45.2	34.4	35.6	37.6	41.2
7 d	50.1	52.1	38.8	40.3	48.8	51.4
28 d	55.6	62.1	43.2	46.2	52.3	60.1

**Table 9:** Deposition efficiency of different substrate concentrations.

Concentration of Substrate (mol/L)	Actual Sediment Yield (g)	Theoretical Sedimentary Yield (g)	Deposition Efficiency (%)
0.2	2	5	40
0.5	6.8	12.5	54.4
1	14	25	56
1.5	14.5	37.5	38.7
2	14.8	50	29.6

## Data Availability

The original contributions presented in this study are included in the article. Further inquiries can be directed to the corresponding authors.
